# Genetic architecture of the maize kernel row number revealed by combining QTL mapping using a high-density genetic map and bulked segregant RNA sequencing

**DOI:** 10.1186/s12864-016-3240-y

**Published:** 2016-11-14

**Authors:** Changlin Liu, Qiang Zhou, Le Dong, Hui Wang, Fang Liu, Jianfeng Weng, Xinhai Li, Chuanxiao Xie

**Affiliations:** 1 Institute of Crop Science, Chinese Academy of Agricultural Sciences, No. 12 Zhongguancun South Street, Haidian District, Beijing, 100081 China; 2 Anhui Agricultural University, Hefei, Anhui Province 230036 China

**Keywords:** Maize, Kernel row number, Specific-locus amplified fragment sequencing, Bulked segregant RNA sequencing

## Abstract

**Background:**

The maize kernel row number (KRN) is a key component that contributes to grain yield and has high broad-sense heritability (*H* ^*2*^). Quantitative trait locus/loci (QTL) mapping using a high-density genetic map is a powerful approach to detecting loci that are responsible for traits of interest. Bulked segregant ribonucleic acid (RNA) sequencing (BSR-seq) is another rapid and cost-effective strategy to identify QTL. Combining QTL mapping using a high-density genetic map and BSR-seq may dissect comprehensively the genetic architecture underlying the maize KRN.

**Results:**

A panel of 300 F_2_ individuals derived from inbred lines abe2 and B73 were genotyped using the specific-locus amplified fragment sequencing (SLAF-seq) method. A total of 4,579 high-quality polymorphic SLAF markers were obtained and used to construct a high-density genetic map with a total length of 2,123 centimorgan (cM) and an average distance between adjacent markers of 0.46 cM. Combining the genetic map and KRN of F_2_ individuals, four QTL (*qKRN1*, *qKRN2*, *qKRN5*, and *qKRN8-1*) were identified on chromosomes 1, 2, 5, and 8, respectively. The physical intervals of these four QTL ranged from 4.36 Mb for *qKRN8-1* to 7.11 Mb for *qKRN1* with an average value of 6.08 Mb. Based on high-throughput sequencing of two RNA pools bulked from leaves of plants with extremely high and low KRNs, two QTL were detected on chromosome 8 in the 10–25 Mb (*BSR_QTL1*) and 60–150 Mb (*BSR_QTL2*) intervals. According to the physical positions of these QTL, *qKRN8-1* was included by *BSR_QTL2*. In addition, *qKRN8-1* was validated using QTL mapping with a recombinant inbred lines population that was derived from inbred lines abe2 and B73.

**Conclusions:**

In this study, we proved that combining QTL mapping using a high-density genetic map and BSR-seq is a powerful and cost-effective approach to comprehensively revealing genetic architecture underlying traits of interest. The QTL for the KRN detected in this study, especially *qKRN8-1*, can be used for performing fine mapping experiments and marker-assisted selection in maize breeding.

**Electronic supplementary material:**

The online version of this article (doi:10.1186/s12864-016-3240-y) contains supplementary material, which is available to authorized users.

## Background

Maize (*zea mays* L.) is a staple crop that plays an important role in satisfying food and feed demands worldwide. Improvement in grain yield is a main objective for maize breeders [1]. The grain yield per unit area can be analyzed by the plant number, ear number per plant, and ear weight. The ear weight can be analyzed by the kernel number per row, kernel row number (KRN), and kernel weight. These traits are quantitatively inherited and influenced by genotypes, environments, and environment-by-genotype interactions. The KRN significantly contributes to grain yield and has the highest broad-sense heritability (*H* ^*2*^) among the three components [2, 3]. Therefore, the investigation into the genetic architecture underlying KRN is helpful not only for understanding the genetic mechanisms underlying ear development but also for improving the maize grain yield.

The KRN is a quantitative trait that is controlled by multiple quantitative trait loci (QTL) [3–5]. Based on diverse bi-parental genetic populations in maize, QTL mapping has been employed to investigate the genetic architecture underlying the KRN [3–6]. In 2014, five QTL for the KRN were detected in both environments using a F_2:3_ population derived from inbred lines Nong513 and K10HEX206 with four rows [7]. Using 325 recombinant inbred lines (RILs) derived from a cross between B73 and SICAU1212, a total of 12 QTL for the KRN were detected across four environments; three of them (*qERN2-1*, *qERN8-1*, and *qERN8-2*) were consistently identified across four environments [3]. Based on 397 F_2:3_ families derived from the inbreds Ye478 and Dan340, a QTL for the KRN that explains 17.9 % of the phenotypic variance was detected on chromosome 7 across seven diverse environments [5]. Using a set of recombinant chromosomes near isogenic lines derived from the inbred line W22 and teosinte (*Zea mays ssp. Parviglumis*), a major QTL explaining ~51 % of phenotypic variance was fine mapped into an interval of 203 kb [4]. Combined with map-based cloning, QTL mapping has been proven to be a powerful approach to exploring the genes or genetic elements that are responsible for the KRN [8, 9]. In 2013, one QTL for the KRN that explains ~8.4 % of phenotypic variance was identified on chromosome 4 using the intermated B73 × Mo17 population. The underlying gene *FASCIATED EAR2* was subsequently cloned, which encodes a CLAVATA receptor-like protein that causes increased inflorescence meristem and the KRN [8]. In 2015, a major QTL for the KRN *KRN4* was fine mapped on chromosome 4; the cloning of this QTL revealed that a 1.2-kb transposon-containing insertion was responsible for increasing the KRN by regulating the expression level of the SBP-box gene *Unbranched3* [9].

Although the QTL for the KRN have been successfully identified via QTL mapping, the low densities of the genetic markers and the complexity of the maize genome caused significant challenges to fine mapping and the exploration of casual genes or elements [10]. The resolution of QTL mapping can be improved using high-density genetic markers due to an increased number of detected recombination events [11, 12]. Advances in next-generation sequencing technologies has caused a rapid decline in the affordable cost of genotyping-by-sequencing (GBS), which is a popular genotyping method that can produce high-density genome-wide markers without prior knowledge [13]. QTL mapping based on GBS has significantly improved the resolution of the QTL detected for different types of quantitative traits [11, 14]. For example, using a genetic map including 6,533 bin markers from low-coverage GBS, *qTBN5* and *qTBN7* for the tassel branch number were detected within small physical intervals of ~800 kb and 1.6 Mb, respectively [11]. Based on a high-density genetic map including 4,932 bin markers, *qLA2a* and *qLOV4* were mapped into small intervals of ~4.30 Mb and ~21.66 Mb, respectively [12]. Using a high-density genetic map containing 4,183 bin markers, *pQTL10* for plant height, ear height, and internode number was detected within an interval of 14.6 Mb [14]. These studies have shown that QTL mapping using a high-density genetic map can accelerate the progress of dissecting the genetic basis of quantitative traits.

In addition to QTL mapping, bulked segregant analysis (BSA) is another strategy that has been employed to rapidly identify genetic determinants underlying phenotypic variation [15]. Based on two groups that show opposing phenotypes of interest traits, the distribution patterns of genome-wide alleles are examined by molecular markers, such as single nucleotide polymorphism (SNP) [16]. A non-random distribution of alleles between two groups likely indicates that this locus is linked with the trait of interest. For example, the co-dominant amplified fragment length polymorphism marker p7m36 was linked to the *rhm* gene for resistance to southern corn leaf blight by using BSA [17]. Recently, the efficiency and power of BSA has been improved by significant advances in next-generation sequencing technologies [18–20]. For example, the use of BSA with next-generation sequencing technology, a novel resistance gene *Pi65(t)* conferring a broad-spectrum resistance to the fungus *Magnaporthe oryzae* was fine mapped within an interval of 1 Mb [20]. Using BSA combined with the specific-locus amplified fragment sequencing (SLAF-seq) strategy, a single dominant gene *PhR10* conferring resistance to the isolate *Byl4* (*race 3*) was fine mapped into an interval of 2.57 Mb [19]. BSA has also been combined with RNA sequencing to rapidly and precisely detect genetic determinants underlying traits of interest [18].

In this study, an F_2_ population was constructed from a cross between inbred line B73 with an average KRN of 16 and inbred line abe2 with an average KRN of four. Using this population, QTL mapping for the KRN was performed based on the high-density genetic map that was constructed via SLAF-seq. In addition, bulked segregant RNA sequencing (BSR-seq) was employed to detect the QTL for the KRN [18]. Our results indicated that combining QTL mapping using a high-density genetic map and BSR-seq was a powerful approach to rapidly fine mapping QTL for traits of interest in maize.

## Methods

### Plant materials

A large F_2_ population was constructed from a cross between inbred lines B73 and abe2. B73 is a non-waxy and yellow endosperm maize with a KRN of 16 ± 2. abe2 is a waxy and white endosperm maize with a stable KRN of four. From the large F_2_ population, 300 F_2_ plants were randomly selected and subjected to GBS using the SLAF-seq strategy [21], which were also employed to investigate the KRN in Yunnan Province in China (21.27°N, 100.25°E). A RIL population consisting of 241 lines were subsequently constructed from these 300 F_2_ plants using a single seed descend method (Additional file 1: Table S1). The KRN of the RILs was investigated using a randomized block design with two repeats in the winter of 2015 in Hainan Province in China (18.20°N, 109.50°E). Based on analysis of variance (ANOVA), the *H* ^2^ for the KRN was calculated as the formula: *H* ^2^ = *σ*_*g*_^2^/(*σ*_*g*_^2^ + *σ*_*e*_^2^), where *σ*_g_^2^ and *σ*_e_^2^ represent the estimated variances for the genetic effects and the random error, respectively [22].

### DNA extraction and genotyping

The genomic DNA of the 300 F_2_ plants and two parents were extracted following the protocol of the Plant Genomic DNA Kit (TIANGEN, Beijing, China). The genomic DNA of the RIL population was extracted from the upper leaves of each RIL using a CTAB procedure [23]. The quality and quantity of the DNA were verified using 1.0 % agarose gels and spectrophotometry (Nanodrop 2000, Thermo Scientific, USA).

The 300 F_2_ plants and two parents were genotyped using the SLAF-seq method [21]. Based on the SLAF pre-design experiment using B73 RefGen_v2 (http://ftp.maizesequence.org/release-5b/), the genomic DNA was digested using the restriction enzyme *Hae*III. The SLAF library was constructed in accordance with the protocol established by Biomarker Technologies Co. Ltd in Beijing, China. Then, selected SLAFs were sequenced using the Illumina HiSeq 2500 pair-end sequencing platform (Illumina, Inc; San Diego, CA, USA).

The RIL population was genotyped using SSR markers, which were downloaded from MaizeGDB (http://www.maizegdb.org/). Vt2 is an insertion/deletion (InDel) marker developed on an InDel between the B73 and abe2 sequences of the gene *vanishing tassel2* [24]*.* The primer sequences of SSR and Vt2 were synthesized by Beijing AuGCT Bio-technology Co. Ltd. PCR amplicons were separated using 8 % polyacrylamide gel electrophoresis and visualized by silver-staining.

### Construction of genetic map and QTL mapping

SLAF pair-end reads with clear index information were grouped into one SLAF locus if the sequence similarity exceeded 90 % as detected using the software BLAT [25] (−tile size = 10, −step size = 5). For each SLAF locus, alleles were defined according to the minor allele frequency (MAF) evaluation because true alleles had significantly higher MAF values than reads containing sequence errors. Clusters with more than four tags or with sequence depth less than 302 were filtered out. SLAFs with two to four tags were considered to be potential polymorphic SLAFs. SLAFs were verified by the alleles origins based on the deep-sequencing of the two parents. Polymorphic SLAFs with a parental genotype of aa × bb and offspring genotypes of ab or miss were used to construct a high-density genetic map (Additional file 2: Table S2). All SLAF markers were grouped with a linkage LOD threshold of 3.0, and the positions and order of grouped markers were arranged using the *est.map* function in the R/qtl package [26]. Combining the KRNs of F_2_ plants, the QTL were identified using the composite interval mapping method implemented in the R/qtl package. The LOD threshold for a significant QTL was determined by 1000 permutations and a *P* value of 0.05 using the *mqmpermutation* function implemented in R/qtl.

For the RIL population, the genetic map was constructed using the software QTL IciMapping version 3.2 (http://www.isbreeding.net). Markers were grouped by a linkage LOD threshold of 3.0 and ordered using the nnTwoOpt algorithm. The ordered markers were rippled by the criteria of the sum of adjacent recombination frequencies. Then, the QTL for the KRN were identified using the inclusive composite interval mapping method implemented in the software QTL IciMapping version 3.2 [27].

### Bulked segregant RNA sequencing

The RNA of 62 plants with extremely low KRNs and 61 plants with extremely high KRNs (Additional file 1: Table S1), which were selected from the large F_2_ population, were extracted from the 30-day leaves of these plants following the protocol of the TRIZOL reagent established by Invitrogen life technologies Co. Ltd. The quality and quantity of RNA were verified using 1.5 % agarose gels and spectrophotometry (Nanodrop 2000, Thermo Scientific, USA). Two RNA bulks were constructed by pooling the RNA of plants with low or high KRNs with equal quantity and sequenced by Data2bio Co. Ltd in Beijing using the Illumina HiSeq 2000 pair-end sequencing platform (Illumina, Inc; San Diego, CA, USA). Raw reads were trimmed using a base PHRED quality value threshold of 15. The remaining nucleotides were then scanned using overlapping windows of 10 bp, and sequences beyond the last window with an average quality value less than the specified threshold were truncated [28].

Clean reads were aligned to B73 RefGen_v3 (www.maizegdb.org) using the software GSNAP [29]. The uniquely mapped reads with less than two mismatches every 36 bp and five bases for every 75 bp as tails were used for subsequent analysis. Polymorphisms were carefully examined and putative heterozygous SNPs were identified based on the pooled RNA-seq data of the two samples in comparison using the following criteria: (1) The first and last three aligned bases of each read were discarded; (2) Each polymorphic base must have a minimum PHRED quality value of 15; (3) The base-pair call must be supported by at least three unique reads; (4) At least 30 % of all aligned reads covering that position support the two most common alleles, and the sum of reads of the two most common alleles account for at least 80 % of all aligned reads covering that nucleotide position. In order to reduce noise, SNPs were retained only if they had at least three reads per allele in the high KRN pool and at least five reads in the low KRN pool. Polymorphic SNPs were used to map the QTL for the KRN using a Bayesian approach [18]. The posterior probabilities were normalized by dividing the maximum linkage observed in a high confidence SNP [18].

## Results

### Genotypes of abe2, B73, and their F_2_ population

A total of 194,886 SLAFs ranging from 414 bp to 444 bp were predicted to randomly distribute across the maize genome with an average adjacent interval of 10.60 kb (Table 1). The SLAFs within repetitive regions were expected to be less than 12.28 %. A subtotal of 5,357,875 reads, 3,129,859 reads, and 28,725,153 reads were generated for B73, abe2, and the F_2_ individuals, respectively. The percentage of bases with quality Q20 was 96.33 % and the GC content was 45.67 %. Based on all high quality pair-end reads, a total of 141,587 SLAFs were defined (Additional file 3: Figure S1a). The average sequence depth of SLAFs in B73 and abe2 was 41.15-fold and 42.08-fold, respectively, and the average sequence depth of the SLAFs in each F_2_ plant was 2.21-fold. Of these 141,587 SLAFs, 26,085 SLAFs (18.42 %) were polymorphic (Additional file 3: Figure S1b), and 504 SLAFs were located in repetitive regions with a percentage of 0.3 %. Among the 26,085 polymorphic SLAFs, 21,693 SLAFs were successfully encoded using the genotypes of two parents according to eight types of coding rules (Additional file 2: Table S2). A total of 21,634 polymorphic SLAFs were uniquely mapped to B73 RefGen_v2 (www.maizegdb.org) (Table 1), and a total of 16,569 SLAFs were successfully encoded according to the type with a parental genotype of aa × bb (Additional file 2: Table S2; Additional file 4: Figure S2).

**Table 1:** SLAFs and genetic maps of ten chromosomes

Chr	No. of expected SLAFs	No. of polymorphic SLAFs	No. of SLAFs	Length (cM)	Average length of interval (cM)	Maximum length of interval (cM)
1	39,815	3,035	646	287.65	0.45	1.43
2	21,393	2,550	546	242.84	0.44	1.39
3	20,395	2,594	578	240.01	0.42	1.6
4	20,890	2,627	593	235.35	0.4	1.44
5	19,448	2,197	467	197.04	0.42	1.56
6	14,870	1,664	348	189.24	0.54	1.66
7	15,552	1,901	352	180.88	0.51	1.73
8	15,403	1,801	336	187.36	0.56	2.09
9	13,813	1,584	348	190.89	0.55	1.69
10	13,307	1,681	365	172.06	0.47	1.27

Note: No. of polymorphic SLAFs indicates the number of polymorphic SLAFs with unique position. No. of SLAFs indicates the number of polymorphic SLAFs used in linkage map construction

### High-density genetic map

**Fig. 1:**
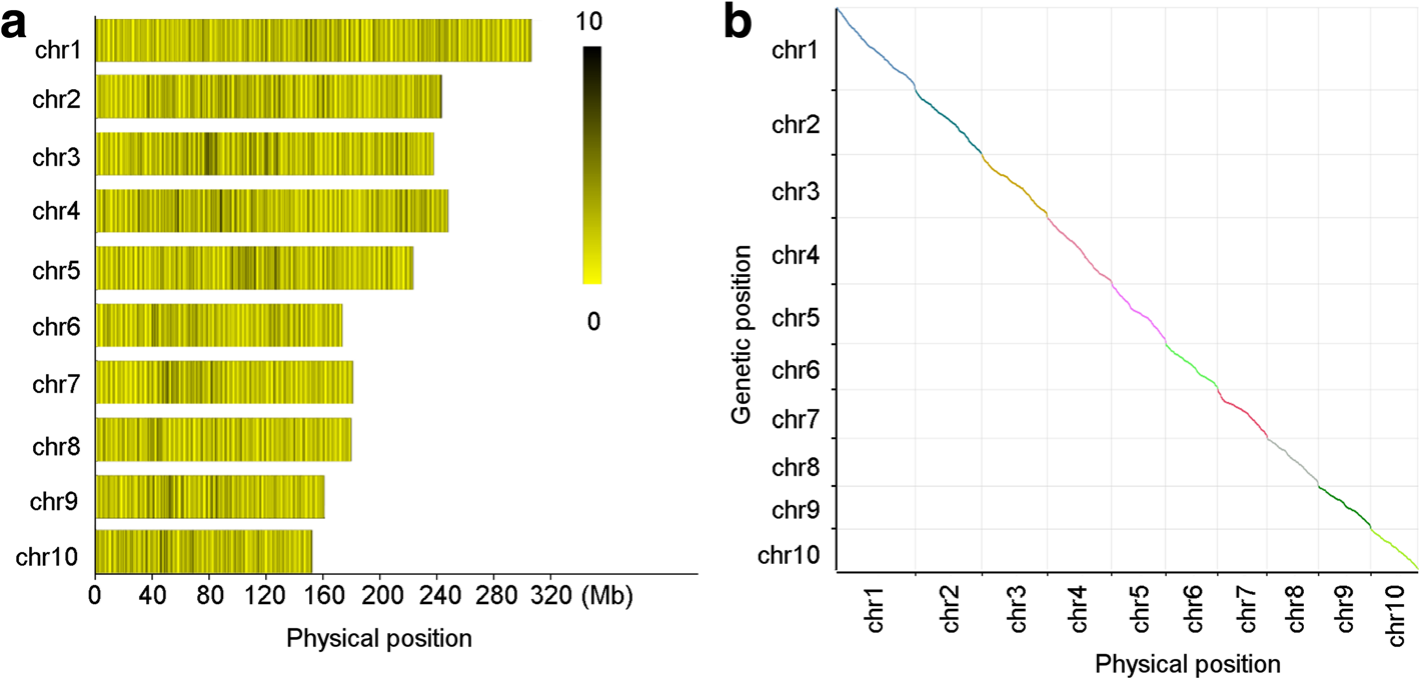
Distribution of polymorphic SLAF markers used to construct genetic map and correlation between genetic position and physical position of markers

Among the successfully encoded 16,569 SLAFs with unique physical position, a total of 4,579 high quality polymorphic SLAFs were employed to construct a genetic map (Table 1, Fig. 1a), which satisfied the following criteria: (1) The sequence depths of SLAFs in B73 and abe2 were larger than 20-fold; (2) the genotype integrity in the F_2_ population was higher than 70 %; and (3) the segregant ratio in the F_2_ population did not significantly differ from 1:2:1 (*P* = 0.05). The average sequence depth of these 4,579 SLAFs in B73 and abe2 was 92.47-fold and 95.42-fold, respectively. No singleton was detected according to the genotypes of the F_2_ individuals (Additional file 5: Figure S3). The missing genotype of each chromosome ranged from 1.69 % to 2.51 % with an average of 2.01 %. The SLAF markers were classed into ten groups with a linkage LOD between any two markers within one group higher than 5.00. The total length of the genetic map was 2,123 centimorgan (cM) with an average distance between adjacent markers of 0.46 cM (Table 1). The maximum distance between adjacent markers was observed on chromosome 8 with a value of 2.09 (Table 1). The Spearman correlations between the genetic positions and physical positions of SLAFs on each chromosome all exceeded 0.99 and were significant at *P* < 0.01 (Fig. 1b).

### QTL mapping using high-density genetic map

**Fig. 2:**
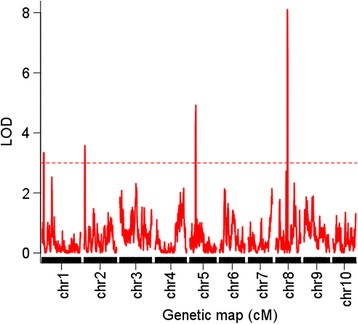
The QTL for the KRN identified in the F_2_ population using a high-density genetic map

According to the LOD threshold value of ~3 as determined by permutation, a total of four QTL were identified on chromosomes 1, 2, 5, and 8 (Table 2) (Fig. 2). The QTL with the largest effect was *qKRN8-1* with a LOD value of 8.01 and explained 15.35 % of the phenotypic variance. *qKRN5* ranked second with a LOD value of 5.08 and explained 15.35 % of the phenotypic variance. *qKRN1* had the smallest effect with a LOD value of 3.29 and explained 3.01 % of the phenotypic variance (Table 2). The QTL alleles from abe2 decreased the KRN. The additive effect for these four QTL ranged from −0.22 to −0.57 with an average value of −1.31, and the dominance effect for these QTL ranged from −0.07 to −2.17 with an average value of −1.15. *qKRN1* had the smallest dominance effect and the largest additive effect. *qKRN8-1* had the smallest additive effect and the largest dominance effect. The physical interval ranged from 4.36 Mb for qKRN8-1 to 7.11 Mb for *qKRN1* with an average value of 6.08 Mb (Table 2). According to the B73 RefGen_v3 gene model (www.maizegdb.org), a subtotal of 178, 163, 55, and 68 protein coding genes were located within *qKRN1*, *qKRN2*, *qKRN5*, and *qKRN8-1*, respectively (Additional file 6: Table S3).

**Table 2:** The QTL for the KRN identified using F2 and RIL population

QTL	Chr	Genetic interval	Physical interval	Physical length (Mb)	LOD	Additive	Dominance	R2 (%)	Population
qKRN1	1	11.11–16.46	268,230,872–275,343,250	7.11	3.29	−0.57	−0.07	3.01	F2
qKRN2	2	9.91–15.66	19,079,906–25,411,626	6.33	3.61	−0.21	−1.11	3.74	F2
qKRN5	5	85.48–91.93	110,426,502–116,922,558	6.51	5.08	−0.31	−1.27	6.06	F2
qKRN8-1	8	86.72–91.44	72,527,096–76,886,956	4.36	8.01	−0.22	−2.17	15.35	F2
qKRN8-1	8	2.35–22.35	16,919,677–129,662,251	112.74	7.43	−0.89		15.52	RIL
qKRN8-2	8	35.01–45.37	156,640,586–161,108,379	4.47	3.95	−0.66		8.25	RIL

### BSR-seq for KRN

A total of 267,884,792 and 247,256,932 paired-end raw reads with a length of 101 bp were obtained in one Illumina HiSeq2000 lane for two RNA pools from extremely high or low KRN plants (Table 3). After trimming, 263,686,133 (98.4 %) and 243,486,618 (98.5 %) clean reads were obtained for the two RNA pools with an average read length of 97 bp. These clean reads of each pool were aligned to B73 RefGen_v3 (www.maizegdb.org) separately, and 217,772,770 (82.58 % of trimmed reads) and 199,145,090 (81.79 % of trimmed reads) uniquely mapped reads were obtained (Table 3). A total of 433,382 SNPs and 175,226 Indels were identified between the uniquely aligned confident reads and the maize reference genome B73 RefGen_v3 (www.maizegdb.org).

**Table 3:** Reads for QTL detection using bulked segregant RNA sequencing

Sample	No. of individuals	No. of raw reads	No. of trimmed Reads (%, trimmed/raw)	Uniquely mapped (%, unique/trimmed)	Average read length (bp)
High KRN	61	267,884,792	263,686,133 (98.43 %)	217,772,770 (82.58 %)	97
Low KRN	62	247,256,932	243,486,618 (98.48 %)	199,145,090 (81.79 %)	97

**Fig. 3:**
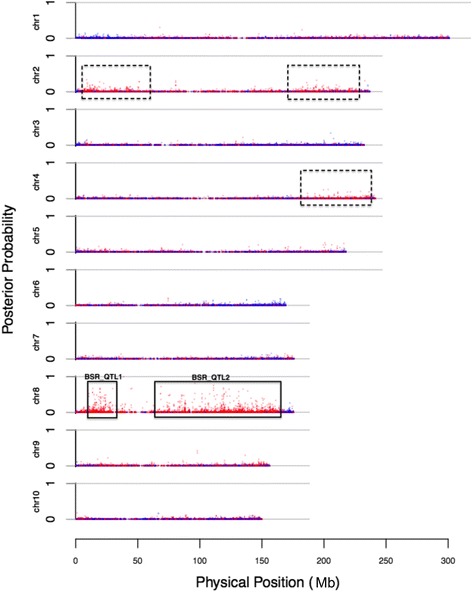
The QTL for the KRN identified using the BSR-seq strategy. The posterior probability of each SNP indicates the probability of complete linkage between the SNP and a causal locus for the KRN

Polymorphic SNPs in the pooled data were used to map the loci for the KRN. After filtering, a total of 202,651 SNPs were employed to determine the probability of the linkage with the causal gene(s) for KRN. Two significant loci were observed in the 10–25 Mb (*BSR_QTL1*) and 60–150 Mb (*BSR_QTL2*) intervals on chromosome 8 (Fig. 3). Three other putative QTL simultaneously exist: two QTL on the short and long arms of chromosome 2 and one QTL on the long arm of chromosome 4 (Fig. 3). According to the physical positions, *qKRN8-1* was included by *BSR_QTL2*.

### Validation of the QTL on chromosome 8 using the RIL population

**Fig. 4:**
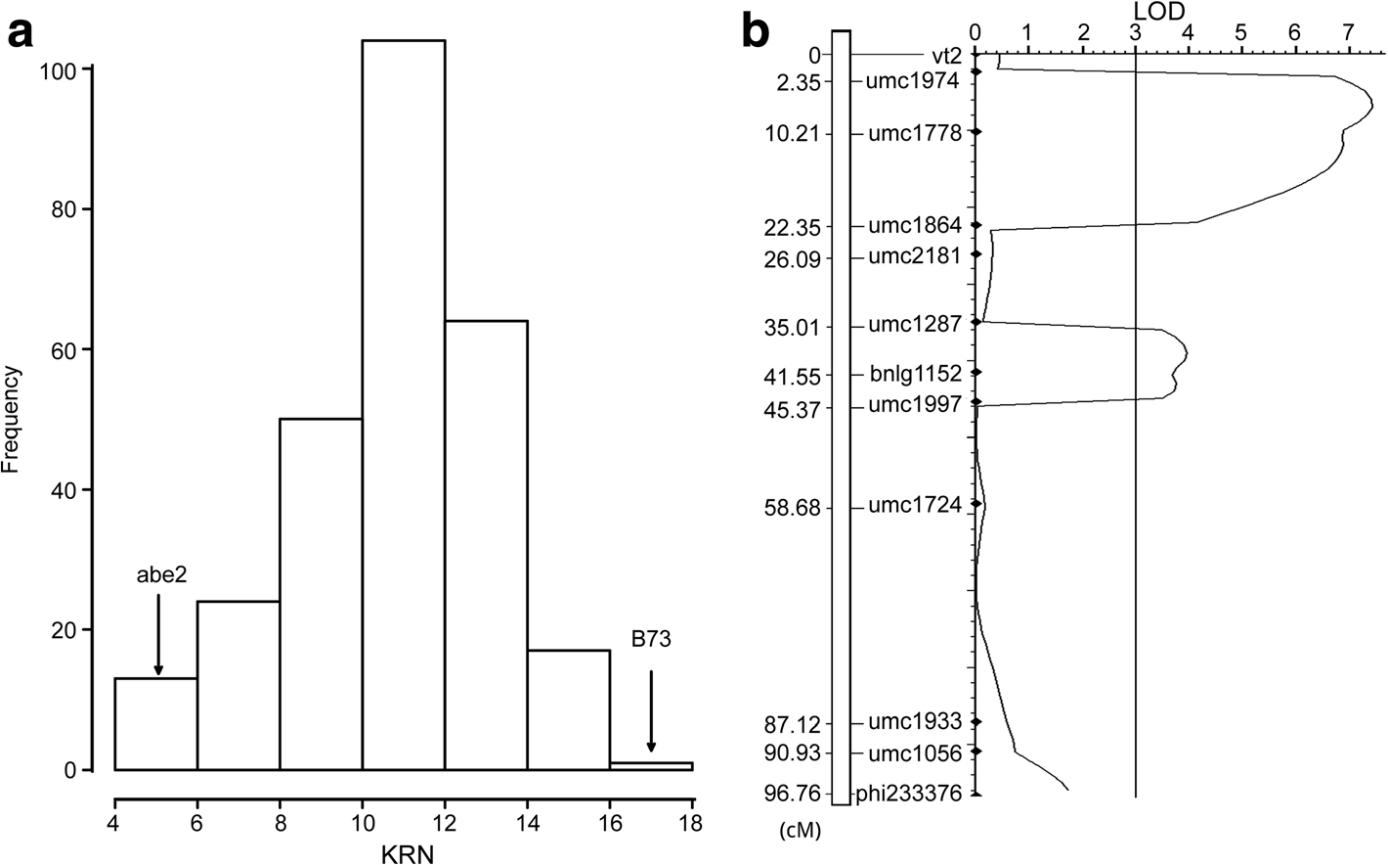
Distribution of the KRN of RILs and the QTL for the KRN detected on chromosome 8 genotype coding method

The KRNs of the RILs ranged from 4 to 18 and normally distributed (Fig. 4a). The KRN difference among the RILs was statistically significant (*P* < 0.01) and the *H* ^*2*^ of the KRN was 0.87 as calculated from the results of ANOVA. A total of 120 markers on chromosome 8 were screened using the genomic DNA of abe2 and B73 and 12 markers (10 %) were polymorphic (Fig. 4b). The length of the genetic map, which was constructed using these 12 polymorphic markers, was 96.76 cM with an average interval of 8.80 cM (Fig. 4b). The maximum interval was observed between umc1933 and umc1724 with a value of 28.44 cM. The order of the markers was consistent with that in IBM 2008 neighbor map (www.maizegdb.org).

Combining the KRNs and the genetic map of the RIL population, two QTL were identified on chromosome 8 with LOD values of 7.43 and 3.95 (Fig. 4b) (Table 1). The QTL which was flanked by SSR markers umc1974 and umc1864 explained 15.52 % of the phenotypic variance and had an additve effect of −0.89 (Fig. 4b) (Table 1). The QTL which was flanked by SSR markers umc1287 and umc1997 explained 8.25 % of the phenotypic variance and had an additive effect of −0.66 (Fig. 4b) (Table 1). According to the physical positions, *qKRN8-1* was identified again and *qKRN8-2* was included by *BSR_QTL2*.

## Discussion

### The power of QTL mapping was increased using a high-density genetic map based on SLAF-seq

QTL mapping is an efficient strategy for exploring the genetic architecture underlying quantitative traits in maize [30, 31]. However, the accuracy and resolution of QTL mapping is significantly affected by the quality and resolution of the genetic map [32]. SLAF-seq is an affordable, rapid, and reliable method for de novo SNP and InDel discovery for large and complex genomes, which combines locus-specific amplification and high-throughput sequencing [21]. It produces thousands of polymorphic markers to generate a high resolution and accurate genetic map. Compared with genotyping using traditional PCR-based markers, SLAF-seq is rapid, cost-effective and does not require prior knowledge of polymorphic information [33–35].

In genotyping using SLAF-seq, the quality of SLAFs plays a key role in the accuracy of de novo SNP or InDel discovery. In this study, the SLAF-efficient selection scheme was developed using B73 RefGen_v2 to improve the efficiency of reduced representation library sequencing [36, 37]. The restricted enzyme *Hae*III was used to digest the genome, and a total of 107,021 SLAFs were predicted. The percentage of SLAFs within repetitive regions was controlled below 12.28 % to simplify the complexity of genomes for facilitating downstream analyses. The copy number variation among fragments is another important factor that affects the quality of SLAFs. To ensure copy number uniformity, a tighter fragment length range (414 bp - 444 bp) was selected to maintain amplification efficiency uniformity during the PCR process. The PCR reaction components and program were also improved to ensure copy number uniformity among fragments. As a result, the average sequence depth of SLAF in B73 (41.15-fold) was close to the average sequence depth of SLAF in abe2 (42.08-fold), and the average sequence depth of SLAF in each F_2_ individual was close to 2.21-fold. The high-density genetic map was constructed using 4,579 high-quality polymorphic SLAFs with an average distance between adjacent markers of 0.46 cM. The Spearman correlation between the marker genetic position and the marker physical position for each chromosome exceeded 0.99 and were significant at *P* < 0.01. This correlation indicated the high quality of the genetic map. Based on the high-density genetic map, a total of four QTL for the KRN were detected. The physical interval ranged from 4.36 Mb to 7.11 Mb with an average value of 6.08 Mb, which were significantly smaller than the physical interval of the QTL identified using traditional PCR-based markers [38, 39]. This result implied the increased power of QTL mapping using a high-density genetic map based on SLAF-seq.

### Comprehensive genetic architecture revealed by combining QTL mapping and BSR-seq

Combining the high-density genetic map and KRNs of 300 F_2_ individuals, a total of four QTL (*qKRN1*, *qKRN2*, *qKRN5*, and *qKRN8-1*) were identified on chromosomes 1, 2, 5, and 8, respectively. Based on reads generated from two RNA pools of plants with extremely opposite KRNs, two significant peaks were observed in the intervals 10–25 Mb (*BSR_QTL1*) and 60–150 Mb (*BSR_QTL2*) on chromosome 8, and three additional putative QTL (two on chromosome 2 and one on chromosome 4) were detected (Fig. 3). These results comprehensively revealed the genetic architecture underlying KRN.

In this study, the F_2_ population was used to map the QTL for the KRN. A comparison of QTL mapping using advanced generation populations, such as RILs [40, 41], indicated that mapping in early-generation populations was rapid and cost-effective. Although the resolution of the QTL was limited by recombination frequency in each F_2_ individual, this resolution may be improved by increasing the population size or the number of polymorphic markers to capture as many recombination events as possible and take full advantage of the linkage information in the F_2_ population [11]. This possibility was proved by our results that the physical intervals of the four QTL ranged from 4.36 Mb to 7.11 Mb with an average value of 6.08 Mb.

The genetic architecture underlying quantitative traits is usually complex [42]. The phenotype results from the genetic architecture and the environment. Although phenotyping with several replications in different environments can reduce the influence of the environment, QTL mapping for the KRN in a single environment is a reasonable approach because the *H* ^*2*^ of the KRN was 0.87 in this study. The estimated *H* ^*2*^ of the KRN was also very hig in other studies [3, 5, 7]. The high *H* ^*2*^ of the KRN indicated that it was not strongly influenced by the environment. In this study, *qKRN8-1* was included by *BSR_QTL2* according to the physical position. It was also validated using QTL mapping with the RILs. Using 325 RILs derived from a cross between B73 and SICAU1212, four QTL for the KRN (*qERN1-2*, *qERN2-1*, *qERN5-1*, and *qERN8-2*) were detected across four environments [3]. *qKRN1* was close to *qERN1-2,* and *qKRN2*, *qKRN5*, and *qKRN8-1* overlapped with *qERN2-1*, *qERN5-1*, and *qERN8-2*, respectively, which were consistently identified across three environments [3]. *qKRN2* and *qKRN5* were also detected by another study. In addition, several studies revealed the QTL for the KRN close to *qKRN2* on the short arm of chromosome 2 [43–45]. These results confirmed the feasible of using QTL mapping for the KRN using F_2_ population and provided confidence in the validity of the QTL.

BSR-seq is an inexpensive and efficient method for gene mapping for mutant phenotypes [18]. It provides not only the position of a gene underlying a mutant phenotype but also patterns of gene expression [46, 47]. In this study, the intervals of two significant QTL for the KRN on chromosome 8 were 15 Mb and 90 Mb. The mapping resolution may be affected by the population size, the accuracy of phenotyping, the pooling strategy and other factors. The potential existence of more than one QTL on chromosome 8 was likely to have significantly impacted the mapping resolution. In addition, the KRN is a quantitative trait [5, 48, 49], and this may result in long intervals of the QTL.

### Fine mapping of the QTL for the KRN

In this study, a total of four QTL (*qKRN1*, *qKRN2*, *qKRN5*, and *qKRN8-1*) for the KRN were identified based on the F_2_ population. According to the B73 RefGen v3 gene model (www.maizegdb.org), a subtotal of 178, 163, 55, and 68 protein coding genes were located within *qKRN1*, *qKRN2*, *qKRN5*, and *qKRN8-1*, respectively (Additional file 6: Table S3). Fine mapping should be conducted to identify casual genes underlying these QTL and facilitate efficient marker-assisted selection. The strategy of the recombinant-derived progeny test was an efficient method for fine mapping [50]. It located the QTL by testing associations between molecular markers and phenotypic variance in progenies derived from recombinants [51, 52]. In addition, the high-density polymorphic markers provided by SLAF-seq and BSR-seq can facilitate fine mapping experiments using additional populations.

According to the LOD and effects, *qKRN8-1* should be priorly investigated because it had the highest LOD and was validated by the BSR-seq and the QTL mapping using the RIL population. A total of 68 protein coding genes were located within *qKRN8-1*, in which five genes have been annotated. However, the expression patterns of genes within *qKRN8-1* from the BSR-seq results were not employed to prioritize candidate genes because no biologically replicated RNA-Seq data existed and the RNA for sequencing was not extracted from the ear where the phenotype was evident. The accurate identification of differentially expressed genes in maize ears with biologically replicated data may facilitate the cloning of genes responsible for the KRN.

## Conclusions

In this study, we proved that QTL mapping for traits with high *H* ^*2*^ is feasible in early-generation populations. Combining QTL mapping using a high-density genetic map and BSR-seq is a powerful and cost-effective approach for the comprehensive dissection of genetic architecture underlying traits of interest. The QTL for the KRN detected in this study, especially *qKRN8-1*, can be employed for performing fine mapping experiments and marker-assisted selection in maize breeding.

## Additional files

Additional file 1: Table S1. KRNs for the F_2_ population, the two pools, and the RIL population. (XLSX 26 kb)
Additional file 2: Table S2. Rules of genotype coding used in genotyping F_2_ population by SLAF-seq. (XLSX 11 kb)
Additional file 3: Figure S1. Distribution of SLAFs obtained by sequencing and polymorphic SLAFs across ten chromosomes. (TIF 1525 kb)
Additional file 4: Figure S2. Recombination map of the F_2_ population derived from inbreds B73 and abe2. The recombination map consists of 4,579 polymorphic SLAF markers. Physical position is according to B73 RefGen_V3. Red: B73 genotype; blue: abe2 genotype; green: heterozygous genotype; white: missing. (TIF 340 kb)
Additional file 5: Figure S3. Distribution of SLAFs encoded using the eight types of genotype coding method.(TIF 3488 kb)
Additional file 6: Table S3. Protein coding genes within four QTL identified in the F_2_ population. (XLSX 37 kb)

## Abbreviations

BSA: Bulked segregant analysis
BSR-seq: Bulked segregant RNA sequencing
cM: Centimorgan
GBS: Genotyping-by-sequencing
*H*^*2*^: Broad-sense heritability
KRN: Kernel row number
MAF: Minor allele frequency
QTL: Quantitative trait locus/loci
RIL: Recombinant inbred line
RNA: Ribonucleic acid
SLAF-seq: Specific-locus amplified fragment sequencing
SNP: Single nucleotide polymorphism
