# Supplementary figures and images for: Genetic architecture of the maize kernel row number revealed by combining QTL mapping using a high-density genetic map and bulked segregant RNA sequencing
Source: BMC Genomics. 2016 Nov 14;17:915. doi: 10.1186/s12864-016-3240-y (PMC5109822; doi:10.1186/s12864-016-3240-y)

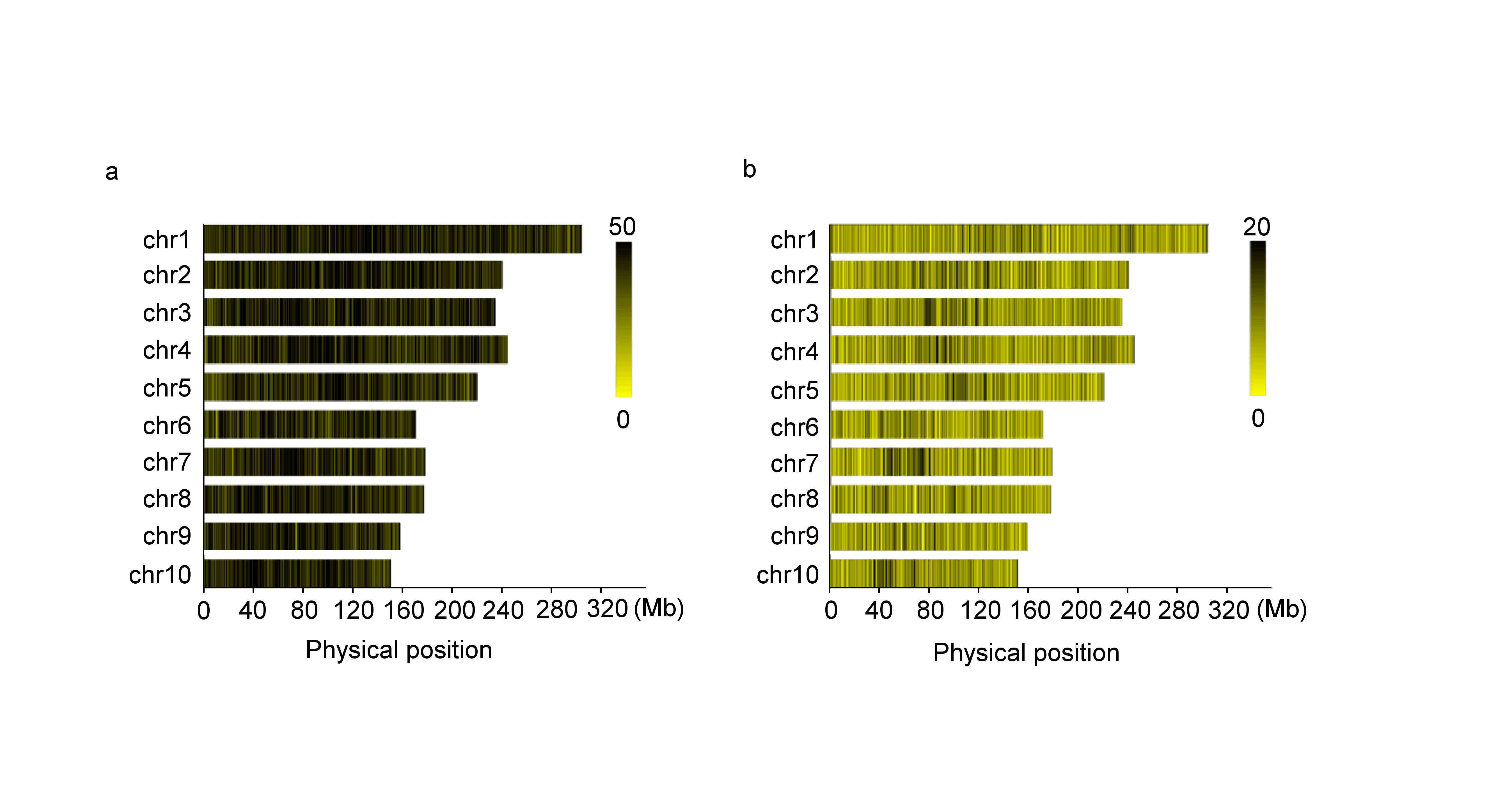

Supplement: Additional file 3: Figure S1. — Distribution of SLAFs obtained by sequencing and polymorphic SLAFs across ten chromosomes. (TIF 1525 kb) [file 12864_2016_3240_MOESM3_ESM.tif]

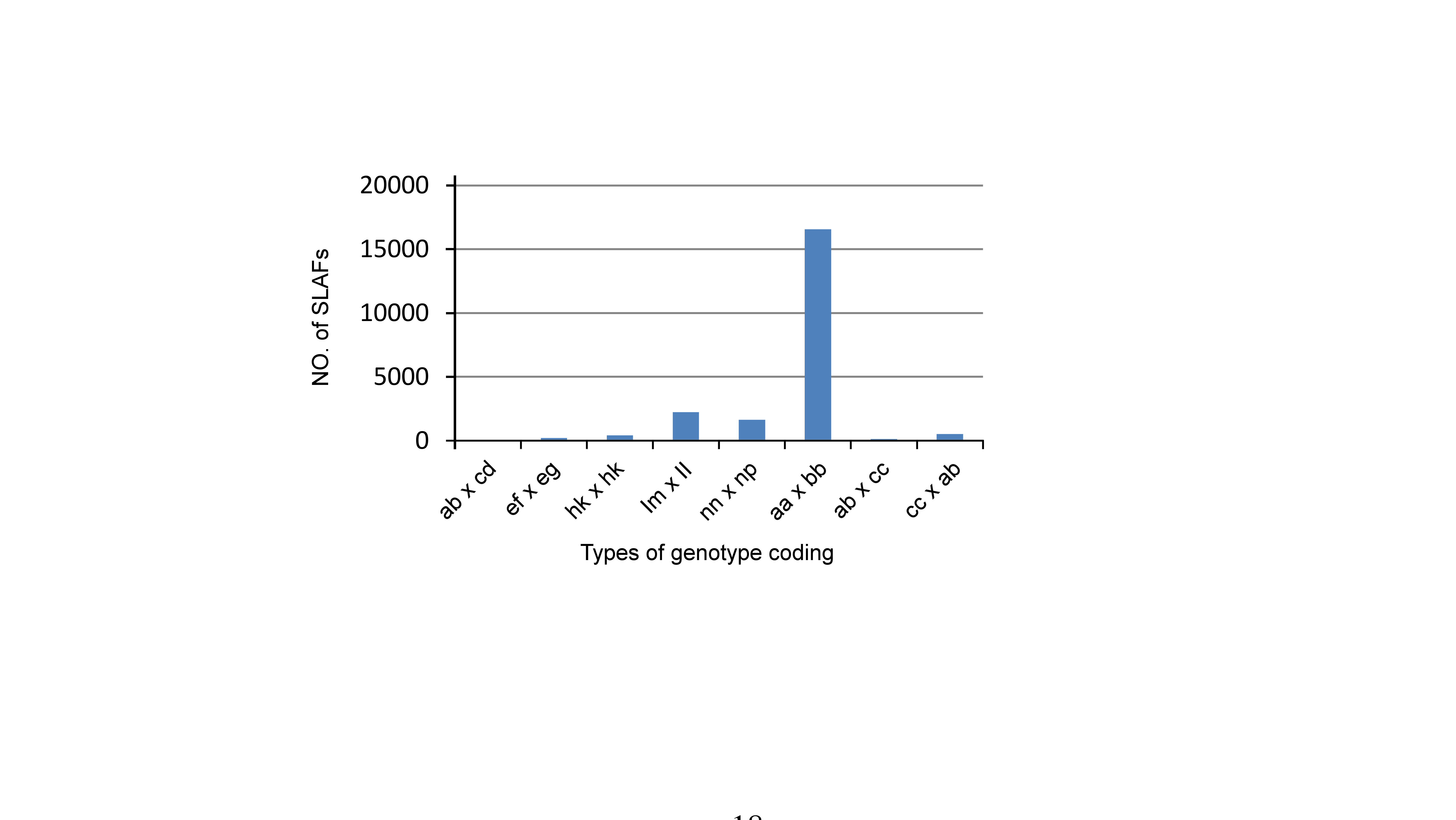

Supplement: Additional file 4: Figure S2. — Recombination map of the F2 population derived from inbreds B73 and abe2. The recombination map consists of 4,579 polymorphic SLAF markers. Physical position is according to B73 RefGen_V3. Red: B73 genotype; blue: abe2 genotype; green: heterozygous genotype; white: missing. (TIF 340 kb) [file 12864_2016_3240_MOESM4_ESM.tif]
